# Listening to the Voice of the Hospitalized Child: Comparing Children’s Experiences to Their Parents

**DOI:** 10.3390/children9121820

**Published:** 2022-11-25

**Authors:** Haneen Ali, Yasin Fatemi, Astin Cole, Sufyan Tahat, Duha Ali

**Affiliations:** 1 Health Services Administration Program, Auburn University, Auburn, AL 36849, USA; 2 Department of Industrial and Systems Engineering, Auburn University, Auburn, AL 36849, USA; 3 351 W Thach Concourse, 7080 Haley Center, Auburn University, Auburn, AL 36849, USA; 4 Public Administration and Public Policy, Auburn University, Auburn, AL 36849, USA

**Keywords:** hospitalized children, patients’ experience, Child HCAHPS

## Abstract

Background: Pediatric patients can provide feedback about their healthcare experiences. However, most do not and are instead represented by their parents. A widely accepted notion is that pediatric patients lack the capacity, vocabulary, and preferences needed to answer meaningful questions related to their healthcare. However, because the pediatric patients’ experience can differ from the perspective of their parents, the use of proxy reporting ultimately reduces the hospital’s ability to address the concerns of pediatric patients directly. Objectives: The overall goals of this survey study were (1) to identify the key domains whereby pediatric patient and parent preferences differ and (2) to investigate the extent to which pediatric patients perceive their healthcare experiences differently from their parents. Methods: This study employed an adult version, an older child version, and a younger child version of a 47-question survey instrument divided into seven key domains: experience with the admission process, interactions with nurses, interactions with doctors, care in the hospital, hospital environment, experience before discharge, and overall score. The instrument was developed to survey children (ages 4–17) recently discharged from the hospital and their parents or guardian. Results: The findings suggest pediatric patients reported interest in engaging with their doctors about their healthcare. However, pediatric patients younger than 13 years old reported having fewer pediatric doctors ask them about their healthcare, with the youngest group reporting the worst experiences. Moreover, differences in perceptions were identified between pediatric patients and their parents regarding hospital quality and performance. Pediatric patients reported less pain and higher satisfaction regarding the cleanliness of the room and their experience in the admission process than their parents. Patient and parent responses were significantly different within each of the seven domains. Conclusion: The results speak to the larger discussion of whether current hospital settings can engage with small children at the level necessary to capture meaningful feedback about their healthcare experience. By providing additional evidence to the contrary, this survey study brings further context to misconceptions regarding pediatric patients’ involvement as crucial actors in the patient-and-family-centered care model.

## 1. Introduction

### 1.1. Patient-Centered Care

Maintaining a human-centered orientation in clinical care, research, training, and governance is critical to the evolution of an effective and sustainable healthcare system [1]. Healthcare quality has traditionally been assessed using patient experience and satisfaction [2,3]. While patient experience reflects independent patient interactions across the continuum of care, patient satisfaction is used to measure the extent to which a hospital meets patient expectations of care [4,5,6]. Embedded within the patient experience model is a focus on individualized care and tailoring services to meet patient preferences and engage them as partners in their care [7]. However, using proxy reporting from a pediatric patient’s parent or guardian can discourage hospital settings from engaging their younger pediatric patients as partners in their healthcare. 

### 1.2. Child Hospital Consumer Assessment of Healthcare Providers and Systems 

The Child Hospital Consumer Assessment of Healthcare Providers and Systems (Child HCAHPS) was developed to be a publicly available standardized survey of the pediatric inpatient experience of care. The purpose of the Child HCAHPS is to benchmark the pediatric inpatient experience across hospitals and improve inpatient care quality [8,9]. Various studies have explored which aspects of a child’s health experience parents tend to get right and wrong when giving experience reports compared with the perspective of their children, who are the actual patients [4,5,6,10,11]. For example, exaggerations of pediatric patient experiences reported by parents filling out surveys for their children have been found, in comparative studies, to overestimate pain scores among children undergoing minor operations [11] and the consequences of children’s illnesses [10]. Such surveys also underestimate the negative experiences of pediatric patients regarding their perceptions of pain relief [5], not being involved in their healthcare [4], and the overall quality of engagement of providers in explaining their healthcare needs [6]. Studies have also successfully collected meaningful feedback from children aged 8–15 years old regarding their healthcare experiences; doing so has yielded crucial information beyond what is possible by collecting the parent’s perspective alone [5,12,13,14,15]. 

**Pediatric patients and feedback.** The primary reason the Child HCAHPS allows parents to answer for their children is that it is assumed that children cannot comprehend the nature of the questions [5,12,13,14,15]. However, the emerging literature on interactive learning programs suggests that the questionnaire would be easier for children to answer if the questions and overall structure reflected the child’s stage of development [5,13,16]. From these insights, a survey was created to test whether pediatric patients report perceiving the quality of their hospital experiences differently from their parents by capturing both the child patient’s and the parent or guardian’s feedback using an interactive learning game based on the pediatric patient’s proximal zone of development [17,18,19]. This paper will report the results of an interactive Qualtrics survey to test the differences in perceived hospital experience between the child patient and their parent. 

Children can provide meaningful feedback concerning their healthcare experiences, including painful procedures [5], chronic diseases [15], and the condition of their internal states, such as emotional distress, fatigue, or gastrointestinal symptoms [13]. More importantly, children perceive their healthcare experience differently from their parents [5,12,13,15,20,21]. However, the current practice of Child HCAHPS surveys of allowing a parent or guardians to answer for the child patient can misrepresent these experiences, leading to inconsistent patient experience data and an inability to distinguish the parents’ preferences from the true preferences of the child patient. Additionally, the practice goes against the goal of patient-and-family-centered care, which seeks to include the family in support of the patient rather than replace the true preferences of the patient with the stated preferences of their parent or guardian [22,23,24]. Efforts to correct this problem of misrepresentation have necessitated the creation of a child-patient experience survey that assesses both the child’s and the parent’s healthcare perspectives [5,12,13,15,20,21]. 

However, challenges in capturing children’s feedback in hospital settings may persist when attempting to capture both the child’s and parent’s feedback. Children need additional support to properly share their healthcare experiences in a way that translates to an actionable item [16,18]. A systematic literature review of child patient experience studies revealed that many reported inconclusive results regarding the child patient’s response [16]. The authors found evidence suggesting that most studies used questionnaires with language too advanced for small children to understand quickly and efficiently [16]. Emerging studies suggest that interactive learning programs can help engage child patients in health literacy by lowering language barriers and connecting the question domains to the child patients’ proximal zone of development [17,18,19]. Using tools that engage pediatric patients at their level of development can enhance the quality of their hospital feedback by reducing barriers to survey engagement related to age. For example, one study used an interactive application—Fabio the Frog (FtF)—to better understand children’s experiences across various ages, abilities, and medical conditions [19]. The authors found that a questionnaire design which uses fun animations to make answering survey questions more appealing to children had nearly the same high internal consistency as a survey of their parents. As a result, this study sought to develop a questionnaire similar to the Child HCAHPS that identifies critical domains of satisfaction in a hospital setting and makes answering the questions more appealing to younger pediatric patients by targeting their current stage of development. Using an interactive study developed in previous work [17], this study seeks (1) to identify the key domains whereby pediatric patient and parent preferences differ and (2) to investigate the extent to which pediatric patients perceive their healthcare experiences differently from their parents. 

## 2. Method

### 2.1. Questionnaire Instrument

Multiple versions of a 47-question interactive survey instrument were administered to investigate the healthcare experience of recently hospitalized children (ages 4–17). The survey consisted of 47 questions. In addition to demographic questions, the survey instrument had seven key domains for a total of 40 questions: experience in the admission process (4 items), care in the hospital (13 items), hospital environment (8 items), interactions with nurses (3 items), interactions with doctors (3 items), experience before discharge (7 items), and overall rating (2 items).

#### 2.1.1. Experience with the Admission Process

Patient perceptions of hospital quality often begin during the admission process. Factors such as the wait time, the quality of the waiting room, and the quality of the intake process can influence patient satisfaction. Experience with the admission process measures how patients and parents perceive the quality of the admission process [3,5,6,10,25,26,27,28]. 

#### 2.1.2. Hospital Environment

The perceived quality of the layout and cleanliness of a hospital room is expected to differ between parent and pediatric patients, as the parent is likely to have more information regarding the true value of a clean space in a hospital setting. The hospital environment domain measures how patients and their parents perceive the quality of the hospital environment [3,18,19,25,26,27,28].

#### 2.1.3. Care in the Hospital

Care in the hospital measures how patients and their parents perceive the quality of the hospital care procedures [3,4,5,25,26,27,28].

#### 2.1.4. Interactions with Nurses

Nurses are the first and most crucial interpersonal connection between patients and healthcare providers. Interactions with nurses measure how nurses are perceived to engage in communication with the pediatric patient and their parent or guardian [3,8,9,25,26,27,28].

#### 2.1.5. Interactions with Doctors

The success of health treatments requires the physician’s communication of the patient’s healthcare treatment and the understanding from the patient on what they must do to achieve their healthcare needs. Interactions with doctors measure how physicians are perceived to communicate with the pediatric patient and their parent or guardian [3,6,8,9,25,26,27,28].

#### 2.1.6. Experience before Discharge

The discharge process is meant to educate patients on the treatment of care they must adopt when they get home, including education about new medications and dietary restrictions. Experience before discharge measures how pediatric patients perceive their engagement and understanding of their treatment before discharge [3,13,25,26,27,28].

#### 2.1.7. Overall Rating

The overall rating measures the overall perception of quality that pediatric patients and parents gave to their hospital experience [3,13,25,26,27,28].

The participants were recruited based on a flyer designed and distributed to the city. In addition, those parents or guardians whose children had recently experienced hospitalization due to health conditions were eligible to attend the study. 

**Figure 1:**
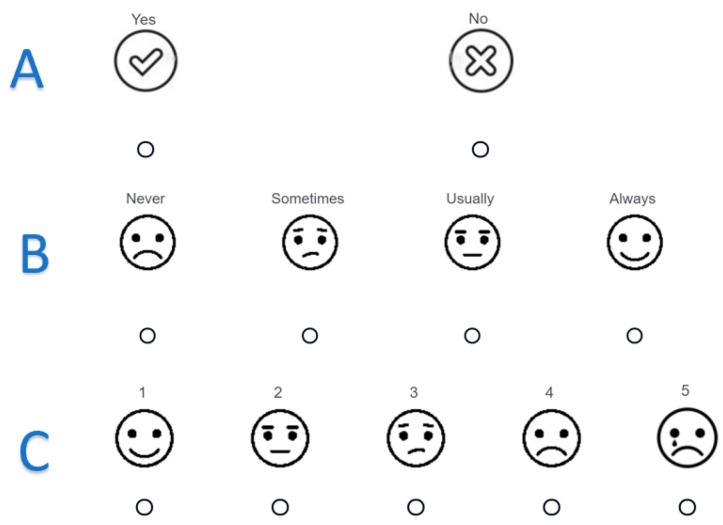
An example of the Likert scale used in the younger children’s version of the survey interface; (**A**): yes/no question, (**B**): never/sometimes/usually/always question, (**C**): 1 to 5 frequency question.

This study’s questionnaire design is based on previous work [12,14,25,26,27,28], the Child HCAHPS [8,9], Press Ganey [27], and input from a wide range of focus groups. This survey was simultaneously administered on two iPads—one for the parents and one for the children—using three versions of the survey tailored to (1) younger children, (2) older children, and (3) parents or guardians. Although each version featured the same questions, the younger children’s version employed a visual Likert scale and audio feature (see Figure 1 for examples) to help improve pediatric patients’ reading comprehension. In addition, the version for older children used vocabulary that required a lower reading level to understand than the parent or guardian version [17].

### 2.2. Interactive Design

**Figure 2:**
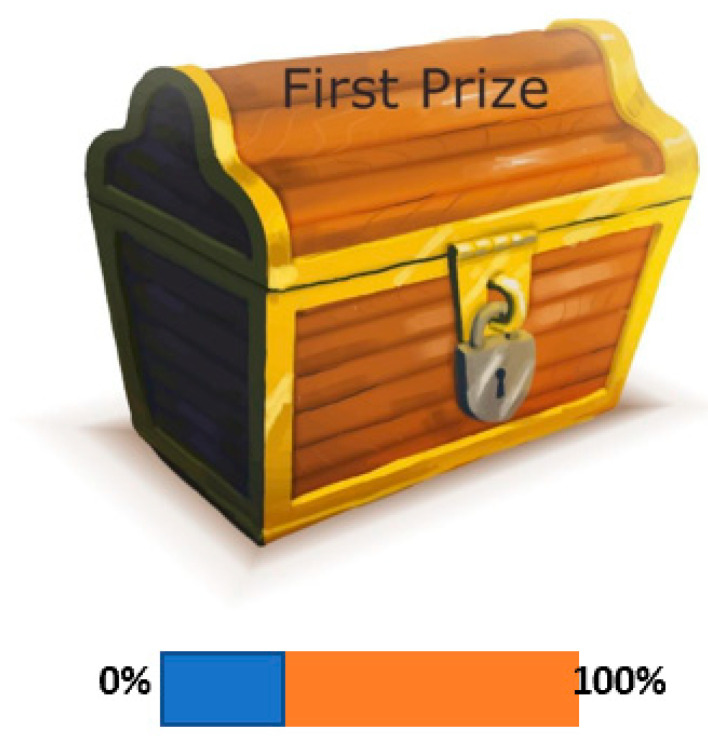
First prize: children pick a prize from the first treasure box, which is smaller and has fewer options than the second one.

The 47 survey questions were organized into seven sections representing a key domain. These sections were further divided into three groups of sections called “levels”: Level 1 (Section 1, Section 2 and Section 3), Level 2 (Section 4 and Section 5), and Level 3 (Section 6, Section 7 and Section 8). Immediately following the completion of each level, the child participant would receive an unexpected tangible reward that corresponds with the completion of said level (see Figure 2). The rewards would increase in size and value with each completed level to reflect the increasingly complex nature of the questions asked. This method was developed to target the participant’s proximal zone of development [29] by helping to motivate the pediatric patient throughout the survey. Rather than focus on the survey length, the reward system incentivized pediatric patients to acknowledge their progress in completing the survey. By providing rewards that correspond with the increasing complexity of the survey questions, the pediatric patient would become engaged in completing each level rather than focusing on the remaining length and time of the questionnaire. 

### 2.3. Ethical Considerations

This study was approved by the university’s Institutional Review Board (18-235 EP 1880). Before participation, each participant and their parent or guardian were given a consent letter to read and sign. Each participant was also compensated USD 20 to account for travel, time, and participation. The pediatric patient’s parent or guardian was encouraged to request where they would prefer to meet with an investigator or volunteer to complete the questionnaire. As a result, the location of the survey sessions varied from reserved spaces in libraries, to their homes, or to reserved spaces at Auburn University when no other locations were recommended.

### 2.4. Reliability and Validity

The instruments used in this study were tested for reliability and validity in a pilot study [17]. The primary purpose of the reliability test was to determine whether the proposed survey questionnaire was too complex or challenging for younger child respondents and whether each proposed domain was appropriate for inclusion. The pilot study conducted preliminary testing of the interview and survey instruments using a sample of locally sourced pediatric patients and parents using the same procedures [17]. Upon the completion of data collection, Cronbach’s alpha was calculated to test the internal reliability of both the parent and child versions of the survey instrument [30,31]. According to Cronbach’s alpha, the internal consistency between domains was within the acceptable range [17].

Prior to pilot testing, the content validity of the survey instrument used for this study was evaluated by a focus group consisting of experts, including two pediatricians, two hospital quality department managers, two patient satisfaction managers, three nurses, two elementary educators, and one nursing faculty member [17]. The initial version, based largely on the wording used in the Child HCAHPS pediatric patient survey, received comments from experts suggesting that the survey instrument was too long and complex for pediatric patients [17]. Therefore, the previous pilot questionnaire and this study’s survey are the results of suggestions by the experts to reduce the number of domain questions to 40 (down from 48) and to adjust the readability of each version to suit the developmental level of the target respondent.

### 2.5. Statistical Analysis

Domain scores were calculated as the average of all relevant domain items [25,26,27]. As only complete survey pairings were included in this study, there are no missing items to account for in this analysis. Data from the survey instrument were processed and analyzed using various approaches. Descriptive statistics were used to examine the general features of the pediatric patient and parent survey scores. A one-way analysis of variance (ANOVA) and the Pearson correlation of the experience scores and domains for pediatric patients and parents, respectively, are provided. ANOVA was used to examine the differences between the given domains along demographic factors. The Pearson correlation was used to test the correlation strength among and between the given domains and the overall rating. Multiple linear regression analysis was used to test the relationship between the overall rating and the given domains. Lastly, the results include a *t*-test to compare the mean of the child patients’ and parents’ replies.

## 3. Results

### 3.1. Descriptive Statistics of Demographic Variables 

Table 1 shows the descriptive analysis of the demographic variables. A total of 44 children ages 5–17 (M = 11, SD = 3.7) were interviewed. The distribution of respondents with respect to gender was relatively even, with 41% (*n* = 18) male respondents and 59% (*n* = 26) female respondents. Nearly 20% (*n* = 8) of the patient respondents were ages 5–7. Patients ages 5–7 took the longest, with an average survey time of 20.59 min, compared with 12.34 min for ages 8–12. Female patients reported the fastest average completion times at 10.7 min. 

### 3.2. Children’s Responses: Age and Gender 

**Experience with the admission process.** The analysis of variance between the age and gender of pediatric patients and the domains tested showed that patients ages 5–7 were significantly more comfortable with the admission waiting area (M = 4.31, SD = 0.35) (*p* = 0.049). However, the ANOVA results between gender and comfort of the waiting area did not reveal any significant differences.

Pediatric patients ages 13–17 reported significantly lower scores for ease of the admission process (M = 3.56, SD = 0.89); male patients reported significantly higher scores (M = 3.89, SD = 0.90). Patients ages 8–12 exhibited the lowest score for the item, “During your admission process, how helpful was the person at the registration desk?” (M = 3.80, SD = 1.11); female patients reported significantly higher scores (M = 4.08, SD = 0.85). 

Lastly, patients ages 5–7 perceived the lowest waiting time in the admission process (M = 4.5, SD = 0.54); male patients reported significantly lower waiting times (M = 39, SD = 1.13). 

**Interactions with nurses.** Along age groups, the ANOVA showed that younger children (<8 years old) reported significantly lower scores for interactions with their nurses (M = 2.75, SD = 0.77) (*p* = 0.045). However, the ANOVA between gender and interactions with nurses did not result in any significant differences. 

Patients aged 8–12 reported significantly higher scores (M = 3.10, SD = 0.85) for the question, “How often did your nurse(s) ask you if you had any questions?” In addition, female patients reported significantly higher scores (M = 2.87, SD = 0.78).

Patients aged 13–17 reported significantly higher scores (M = 3.63, SD = 0.50) for the question, “How often did you feel like your nurse(s) listened to what you had to say?” In addition, female patients reported significantly higher scores for this question (M = 3.59, SD = 0.65).

**Additional domains.** The ANOVA between age and gender and interactions with nurses, care in the hospital, the hospital environment, experience before discharge, and the overall rating *did not* yield any significant differences. 

### 3.3. Correlation Analysis

Table 2 was used to identify significant relationships between the domains of pediatric patient responses.

As shown in Table 2 above, interactions with nurses and interactions with physicians were found to have a positive and significant relationship (*r* [42] = 0.71, *p* = 0.000). Additionally, the admission process experience, interactions with nurses and with physicians, and experience before discharge all have significant and positive relationships with care in the hospital (*r* [42] = 0.58, *p* = 0.00; *r* [42] = 0.41, *p* = 0.005; *r* [42] = 0.50, *p* = 0.001; and *r* [42] = 0.36, *p* = 0.015, respectively). In addition, the experience before discharge was found to have a significant and positive relationship with experience with the admission process (*r* [42] = 0.40, *p* = 0.008), interactions with doctors (*r* [42] = 0.36, *p* = 0.015), and the hospital environment (*r* [42] = 0.53, *p* = 0.000). Lastly, the overall rating from pediatric patients has a significant and positive relationship with the admission process experience (*r* [42] = 0.43, *p* = 0.004), interactions with nurses (*r* [42] = 0.59, *p* = 0.000), care in the hospital (*r* [42] = 0.39, *p* = 0.009), hospital environment (*r* [42] = 0.36, *p* = 0.017), and experience before discharge (*r* [42] = 0.47, *p* = 0.001). 

Table 3 shows the Pearson correlations between the domains of parent and guardian responses. According to Table 3, interactions with physicians have a significant and positive relationship with interactions with nurses (*r* [42] = 0.77, *p* =0.000), care in the hospital (*r* [42] = 0.51, *p* = 0.000), experience before discharge (*r* [42] = 0.51, *p* = 0.000), and overall rating (*r* [42] = 0.52, *p* = 0.000). Additionally, the results show that experience before discharge was found to have a significant and positive relationship with interactions with nurses, care in the hospital, and the hospital environment (*r* [42] = 0.66, *p* = 0.000; *r* [42] = 0.50, *p* = 0.001; and *r* [42] = 0.35, *p* = 0.021, respectively). Lastly, the overall rating ratings of parents and guardians were found to have a significant and positive relationship with interactions with nurses (*r* [42] = 0.55, *p* = 0.000), experience before discharge (*r* [42] = 0.67, *p* = 0.000), and care in the hospital (*r* [42] = 0.58, *p* = 0.000).

### 3.4. Difference between Children and Parents’ Responses

Paired *t*-test was used to compare the mean of the two populations: pediatric patients and parents or guardians. Differences between the reported answers of pediatric patients and parents were tested; α=0.05  for the six domains and the overall rating.

**Experience with the admission process.** The analysis showed a significant difference between the children’s and parents’ responses along the experiences with the admission process domain (*p* = 0.000), with children reporting a higher mean (M = 3.79, SD = 0.69). Over 88% (*n* = 39) of pediatric patients reported being comfortable with the admission process, compared with 77% of the parents and guardians (*n* = 34). Additionally, 82% (*n* = 36) of children reported that the waiting area was comfortable; on the other hand, 77.5% (*n* = 34) of parents agreed that the waiting area was satisfactory.

**Interactions with nurses.** Children (M = 3.24, SD = 0.63) expressed significantly higher satisfaction with nurses than their parents (M = 2.88, SD = 0.68) along the interactions with nurses’ domain (*p* = 0.008). Only 54.5% (*n* = 24) of the patients reported positive experiences with nurses, compared with 77.5% (*n* = 34) of parents. Additionally, 86.5% (*n* = 38) of the children reported that nurses listened to their comments and what they had to say, compared with 63.5% (*n* = 28) of the parents. Ninety-one percent (*n* = 40) of the children reported that they “usually” or “always” understood what the nurses were saying, compared with 72% (*n* = 32) of the parents. Lastly, 59% (*n* = 26) of the children and parents reported that the nurses “usually” or “always” asked them if they had questions.

**Interactions with doctors.** Patients perceived significantly more positive experiences in interacting with doctors (M = 3.31, SD = 0.64) than their parents (M = 2.96, SD = 0.77), (*p* = 0.022). Over 86% (*n* = 38) of the patients reported positive interactions with doctors, compared with 59% reported by parents (*n* = 26). Additionally, over 86% (*n* = 38) of the children understood the physicians’ instructions, compared with over 63% (*n* = 28) of the parents. While 63.5% (*n* = 28) of the children’s responses showed that physicians often discussed their condition, 77.5% (*n* = 34) of the parents reported that doctors talked to them about it. Lastly, 91% (*n* = 40) of the children agreed that doctors “usually” or “always” listened to what they had to say, while 77.5% (*n* = 32) of the parents reported the same.

**Care in the hospital.** The results indicated significant differences between patient and parent responses (*p* = 0.000). The patients reported higher scores related to the hospital’s quality of care than their parents (M = 3.21, SD = 0.31 and M = 2.91, SD = 0.47, respectively).

Over 77% (*n* = 34) of the patients reported that care in the hospital was generally efficient, compared with only 50% (*n* = 22) of the parents. Roughly 91% (*n* = 40) of the patients reported that the staff responded to their requests, compared with only 41% (*n* = 18) of the parents. Additionally, 50% (*n* = 22) of the patients reported that physicians and nurses taught them how to take new medicines, compared with roughly 68% (*n* = 30) of the parents.

Only 63.5% (*n* = 28) of the patients reported that their nurses and doctors explained the side effects of their medicines, compared with 32% (*n* = 14) of the parents. Over 91% (*n* = 40) of the patients reported that they understood what they were told, compared with 41% (*n* = 18) of the parents. Over 45% (*n* = 20) of the patients reported a high level of pain (>3), compared with 100% (*n* = 44) of the parents. Lastly, over 86% (*n* = 38) of the patients reported that nurses “usually” or “always” asked them about their pain, compared with 36.5% (*n* = 16) of the parents.

**The hospital environment.** This study found a significant difference between the patient (M = 2.85, SD = 0.51) and parent (M = 2.13, SD = 0.57) responses for the hospital environment domain (*p* = 0.000).

Over 45% (*n* = 20) of the patients reported positive scores for the comfort of the hospital environment, compared with only 11% (*n* = 5) of the parents. Additionally, 50% of the patients (*n* = 22) reported positive scores for the cleanliness of the room, compared with only 13% (*n* = 6) of the parents. Lastly, 50% (*n* = 22) of the patients reported that three types of entertainment were supplied; the measure for the parents was 56% (*n* = 25).

**Experience before discharge.** The responses of the parents (M = 2.66, SD = 0.41) and children (M = 3.12, SD = 0.34) regarding their experience before discharge were significantly different (*p* = 0.002).

Only 50% (*n* = 22) of the patients reported positive scores for their experience before discharge, compared with 27% (*n* = 12) of the parents. Roughly 59% (*n* = 26) of the patients reported that someone talked to them about any new medicine, compared with 54.5% (*n* = 24) of the parents. Over 72% (*n* = 32) of the patients reported that one of the doctors or nurses taught them how to take new medicines, compared with 63.5% (*n* = 28) of the parents. Additionally, roughly 59% (*n* = 26) of the patients recalled their discharge date, compared with 72.5% (*n* = 32) of the parents. Lastly, over 72% (*n* = 32) of the patients reported that hospital staff explained health problems to look out for, compared with only 68% (*n* = 30) of the parents.

**Overall rating.** The overall rating responses of the patients (M = 7.64, SD = 1.71) and parents (M = 6.45, SD = 1.66) were significantly different (*p* = 0.000). Roughly 86% (*n* = 38) of the patients reported positive scores for the quality of the hospital, compared with 72.5% (*n* = 32) of the parents. Lastly, roughly 82% (*n* = 36) of patients reported that they would recommend their hospital to others, compared with only 59% (*n* = 26) of the parents.

Table 4 and Table 5 provide two multiple linear regression analyses to determine which domains affect the response variables: (1) regression analysis between the child respondents and their overall rating, and (2) regression analysis between the parent respondents and their overall rating.

Table 5 shows that care in the hospital (*b* = 1.41, *p* = 0.021), the hospital environment (*b* = −0.825, *p* = 0.034), and experiences before discharge have a significant and positive effect on the overall score, holding all other variables constant (*b* = 1.51, *p* = 0.000).

Table 4 shows that the variables experience with the admission process (*b* = 0.98, *p* = 0.001), interactions with nurses (*b* = 2.47, *p* = 0.000), and the hospital environment (*b* = 0.726, *p* = 0.042) were found to have a significant and positive effect on the pediatric patients’ overall score, holding all other variables constant. Conversely, interactions with doctors (*b* = −1.56, *p* = 0.000) were found to have a significant and negative effect on the patient’s overall rating, holding all other variables constant.

## 4. Discussion

Although the literature regarding the need to engage children in their healthcare is growing, many studies suggest that younger children are not often thought of as capable of understanding their current health needs.

Table 1 reports that female participants could complete the survey much faster than male participants. Differences in completion times between gender groups might suggest differences in levels of attention and concentration between male and female pediatric patients with respect to age. Future designs will attempt to narrow differences in completion time that variations in attention span might cause by employing additional attention-retaining techniques, such as corresponding sounds and animations, that reward participation between groups of responses. Additionally, it was found that 100% of the questionnaire participants (*n* = 44) understood the cause of their hospitalization. These results suggest that hospitalized children as young as four years old can understand and convey their current health status; however, few studies have engaged with children this young about their healthcare needs.

Differences in health communication among pediatric patients might explain why this study’s one-way ANOVA results in Table 2 and Table 3 suggest that younger patients in this study (ages 4–7) have reportedly worse experiences with respect to perceived care, experiences with nurses, and experiences with doctors, compared with other age groups. Additionally, all three age groups (4–7, 8–12, 13–17) reported significantly different responses for the before-discharge experience with respect to age, with the average response at its lowest for the youngest group and the highest for the oldest group. Healthcare providers trained to communicate with their patients at various development levels are more likely to engage in more effective patient–physician communication [32,33,34,35]. Poor communication between physicians and patients can worsen the overall experience of younger patients, whose fears, anxieties, confusion, or discomforts surrounding their hospitalization are never properly addressed [33,35].

A lack of studies to properly identify the perspective of hospitalized children might explain why so few studies discuss the differences in perspectives between various age groups. For example, this study found that younger patients had a significantly higher comfort level with the admission process than older child patients. Moreover, this study’s Pearson correlation analysis in Table 4 shows that age negatively affects comfort level regarding the admission process. As pediatric patients age, they are more likely to assist in the admission process. The difference in the comfort level that can be observed to increase along age groups might suggest that older children perceive greater barriers in the admission process due to their greater involvement in completing the process.

Lastly, differences between the pediatric patients and their parents were found along the tested domains in Table 4 and Table 5. Among child patient perceptions, while significant and negative trends were found between experiences with doctors and overall rating, significant and positive trends between experiences with the admission process and experiences with nurses and the pediatric patients’ overall rating persisted. Parent perceptions, by comparison, were more focused on the hospital’s processes, with care in the hospital and experiences before discharge having a significant and positive trend with the overall rating and with the hospital environment having a significant and negative trend with the overall rating. These findings provide greater evidence for the results of previous studies that suggest that pediatric patients are more satisfied with quality indicators related to the overall layout and processes of a hospital [16,17]. For example, most child respondents believed their wait time was “short” or “very short” compared with less than one-quarter of the parents. Similarly, pediatric patients were more satisfied with their rooms’ cleanliness than their parents. These findings suggest that in allowing parents to answer for their children, surveys such as the Child HCAHPS allow parents to express their preferences through their child’s voice [5,17].

Pediatric patients also reported significantly lower pain levels than their parents. As parents cannot measure their child’s pain precisely, many parents would likely prefer to exaggerate rather than minimize their child’s level of discomfort. These results are consistent with other studies finding that parents report significantly higher pain levels than their children [10,11].

The findings related to pediatric patient experiences with doctors suggest that pediatric patients reported desiring more patient–physician interactions. However, patients also reported high scores on the item, asking to what extent they felt that their doctors gave more attention to their parents. Overall, experiences with doctors are reported as primarily positive; however, as previously discussed, doctors receive significantly lower scores from the youngest group of pediatric patients and significantly higher scores from the oldest group regarding communication. Furthermore, one-way ANOVA suggests that the youngest groups reported having the worst experiences of all age groups with their doctors. This study’s results suggest that while pediatric patient perceptions of hospital experiences are overall positive, poor physician–patient communication with pediatric patients under 13 years old is likely to contribute to negative trends in the relationship between experiences with doctors and overall rating. These results are consistent with studies suggesting that pediatric doctors often underestimate the ability of younger pediatric patients to provide meaningful feedback [35. One study found that intervention in the form of additional communication training improved a physician’s ability to build relationships with the child patient’s parents and tended to enhance performance in exploring parents’ problems [35]. A more recent study closely examined caregivers’ expectations of physician communication in hospital settings [34]. Most notably, neither work provided evidence to suggest that the pediatric patient’s expectations and feedback regarding their experience were considered in equal measure with that of their parent or guardian. Thus, progress regarding physician–patient communication has likely not reached the point where the pediatric patient’s feedback is a priority in hospital settings. This necessitates further research to bridge the gaps in reliably capturing the experiences of hospitalized children as pediatric patients with meaningful feedback to share.

## 5. Limitations

Although this study’s design can distinguish differences between child and parent, it cannot currently examine differences conditioned on the patient’s English language proficiency. There are likely differences in how child patients of different cultural groups experience hospital settings. For example, one study found that most pediatricians reported using family members to communicate with patients with limited English proficiency (LEP) [32,36]. This likely suggests that the issues and concerns of using parents as the proxy for pediatric patient experiences might be exacerbated among groups associated with LEP. Additionally, this study did not consider clinical factors such as comorbidities or patient acuity when collecting the sample of hospitalized children. Whether a pediatric patient lives with a chronic illness or was hospitalized due to a complication with a pre-existing illness can influence the responses of the patient as well as their parent or guardian’s ability to act as their proxy response. As a result, future studies would benefit from clinical factors and an ethnographic framework to capture the intersectional challenges in capturing pediatric patient feedback. Lastly, without a large and robust sample size, the differences between patient and parent responses could be widened due to differences related to language and literacy barriers.

## 6. Future Work

Planned future work related to this study will use a large, robust sample of diverse pediatric patients and parents to test the implications of language proficiency barriers as obstacles to pediatric patient experience and feedback. This future design will include a Spanish-language version of the surveys currently developed. Additionally, smart apps with animated features and a storybook component will be developed as an ensemble to bolster the current questionnaire design. This medium aims to give child patients under 13 years old a host of characters, themes, and narratives that teach and explain the elements of hospital settings in a manner that holds their attention and encourages patient–physician communication.

## 7. Conclusions

This study found that differences exist between a pediatric patient and their parent regarding their perceptions of a hospital’s quality and performance. Additionally, differences exist between the age groups of pediatric patients, which suggests that younger children receive less attention than older children from their nurses and doctors. These results speak to the larger discussion of whether small children can provide meaningful feedback about their healthcare. By providing additional evidence that they can, this survey study brings further context to misconceptions regarding pediatric patients’ involvement in their own hospitalized care.

## 8. Declarations

### Availability of Data and Materials

The datasets analyzed during the current study are available from the corresponding author on reasonable request.

## Figures and Tables

**Table 1:** Descriptive statistics.

Age	Respondents N	Percent %
5–7	8	18
8–12	20	46
13–17	16	36
Gender	Respondents N	Percent %
1 = F	26	51
2 = M	18	49
Age	Survey Duration M	SD
5–7	20.59	5.59
8–12	12	2.03
13–17	9.34	1.70
Gender	Survey Duration M	SD
1 = F	10.7	2.24
2 = M	15.3	6.30
Note. *N* = 44		

**Table 2:** Pearson correlation analysis—children’s responses.

	M ^a^	SD ^b^	1	2	3	4	5	6	7
1. Experience with the Admission Process	3.79	0.69	—						
2. Interactions with Nurses	3.24	0.63	0.09	—					
3. Interactions with Physicians	3.31	0.27	0.24	0.71 **	—				
4. Care in the Hospital	3.21	0.31	0.58 **	0.41 **	0.50 **	—			
5. Hospital Environment	2.85	0.52	0.136	0.06	0.09	0.142	—		
6. Experience before Discharge	2.82	0.33	0.40 **	0.28	0.36 *	0.68 **	0.53 **	—	
7. Overall Rating	7.63	1.71	0.43 **	0.59 **	0.22	0.39 **	0.36	0.47 **	—

* *p* < 0.05. ** *p* < 0.01. ^a^ Mean. ^b^ Standard Deviation.

**Table 3:** Pearson correlations for parent domain responses.

Variable	M ^a^	SD ^b^	1	2	3	4	5	6	7
1. Experience with the Admission Process	3.29	0.69	—						
2. Interactions with Nurses	2.88	0.63	0.20	—					
3. Interactions with Doctors	2.96	0.27	0.18	0.77 **	—				
4. Care in the Hospital	2.91	0.31	0.22	0.55 **	0.51 **	—			
5. Hospital Environment	2.12	0.52	0.16	−0.08	−0.07	0.20	—		
6. Experience before Discharge	2.39	0.34	0.10	0.66 **	0.51 **	0.5 **	0.35 **	—	
7. Overall Rating	6.45	1.66	0.21	0.55 **	0.52 **	0.58 **	0.05	0.67 **	—

Note. ** *p* < 0.01. ^a^ Mean. ^b^ Standard deviation.

**Table 4:** Multiple linear regression analysis between the child respondents and their overall rating.

Effect	Coefficient	SE Coefficient	*T*-Value	*p*-Value
Constant	−3.10	1.78	−1.74	0.09
Experience with the Admission Process **	0.98	0.283	3.43	0.001
Interactions with Nurses **	2.47	0.344	7.20	0.000
Interactions with Doctors **	−1.56	0.353	−4.43	0.000
Care in the Hospital	−0.014	0.680	−0.02	0.98
Hospital Environment *	0.726	0.344	2.11	0.042
Experience Before Discharge	0.772	0.59	1.29	0.203

Note. * *p* < 0.01, ** *p* < 0.05.

**Table 5:** Multiple linear regression analysis between the parent respondents and their overall rating.

Effect	Coefficient	SE Coefficient	*T*-Value	*p*-Value
Constant	0.79	1.39	0.57	0.575
Experience with the Admission Process	0.381	0.300	1.27	0.212
Interactions with Nurses	−0.659	0.503	−1.31	0.199
Interactions with Doctors *	0.38	0.357	1.07	0.294
Care in the Hospital *	1.41	0.474	2.41	0.021
Hospital Environment *	−0.825	0.376	−2.20	0.034
Experience before Discharge **	1.51	0.369	4.10	0.000

Note. * *p* < 0.01, ** *p* < 0.05.

## Data Availability

The datasets generated during the current study are not publicly available but are available from the corresponding author upon reasonable request.
